# Investigating physicians’ views on non-formulary prescribing: a qualitative study using the theoretical domains framework

**DOI:** 10.1007/s11096-023-01616-7

**Published:** 2023-07-15

**Authors:** Zachariah Nazar, Moza Al Hail, Samaher Al-Shaibi, Tarteel Ali Hussain, Nada Nabil Abdelkader, Abdulrouf Pallivalapila, Binny Thomas, Wessam El Kassem, Yolande Hanssens, Ahmed Mahfouz, Cristin Ryan, Derek Stewart

**Affiliations:** 1 College of Pharmacy, QU Health, Qatar University, Doha, Qatar; 2 Women’s Wellness and Research Center, Hamad Medical Corporation, Doha, Qatar; 3 Hamad Medical Corporation, Doha, Qatar; 4 School of Pharmacy and Pharmaceutical Sciences, Trinity College Dublin, Dublin, Ireland; 5 Pharmacy department, Mohammed Al-Mana College for Medical Sciences, Dammam, Saudi Arabia

**Keywords:** Attitude of health personnel, Behavior change, Clinical pharmacy, Drug safety, Formulary, Inappropriate prescribing, Qualitative research, Theoretical domains framework

## Abstract

**Background:**

Well-designed and well-maintained drug formularies serve as a reliable resource to guide prescribing decisions; they are associated with improved medicine safety and increased efficiency, while also serving as a cost-effective tool to help manage and predict medicine expenditure. Multiple studies have investigated the inappropriate prescribing of non-formulary drugs (NFDs) with statistics indicating that up to 70% of NFD usage being inappropriate or not following the ascribed NFD policies.

**Aim:**

To explore physicians’ views and influences on their prescribing of non-formulary drugs.

**Method:**

Data collection and analysis were underpinned using the Theoretical Domains Framework (TDF). Thirteen semi-structured interviews were conducted within Hamad Medical Corporation, the main provider of secondary and tertiary healthcare in Qatar, with physicians who had submitted a NFD request in the preceding 12 months.

**Results:**

Three overarching themes were identified: *providing evidence-based care for individual patients*; *influences of others*; and *formulary management issues*. Subthemes were mapped to specific TDF domains: *environmental context and resources; social influences; professional role and identity; beliefs about consequences; goals; intentions.*

**Conclusion:**

The behavioral influences identified in this study can be mapped to behavior change strategies facilitating the development of an intervention to promote appropriate prescribing of NFDs with implications for medicine safety and healthcare efficiency.

## Impact statements

- Physicians prescribing non-formulary drugs is linked to a number of the theoretical domains framework behavioral domains: *environmental context and resources; Social influences; Professional role and identity; Beliefs about consequences; Goals; Intentions*.
- Despite physicians’ acknowledgement of the importance of adhering to formulary prescribing, physicians continue to prescribe non-formulary drugs indicating the necessity to prioritize patient care and their belief that as experts in their field, their decisions are justified.
- Educating and communicating to stakeholders including physicians, patients and their carers, on the formulary management is warranted to mitigate against the pressures placed on physicians to prescribe non-formulary medications and increase awareness of its significant relevance.

## Introduction

Drug formularies serve as a reliable resource to guide prescribing decisions and medicine utilization [1–5]. They represent an up-to-date list of the most appropriate, cost-effective and evidence-based medicines indicated for specific disease areas [4–8]. Inclusion in formularies is determined based on efficacy, safety, treatment cost, or cost-effectiveness; and supplemented using the clinical judgment of physicians, pharmacists and other experts in diagnosis and treatment of disease [9–11]. As such, the World Health Organization (WHO) advocates that healthcare institutions implement robust structures and processes to establish and update drug formularies, and advise on the appointment of a drug and therapeutics committee to oversee drug inclusion and set local policies [12].

The international published literature also supports the utility of drug formularies. Studies indicate that well-designed and well-maintained drug formularies are associated with improved medicine safety and increased efficiency, while also serving as a cost-effective tool to help manage and predict medicine expenditure [13–15]. However, it is impossible that a drug formulary will cover every clinical scenario, therefore it is recommended that policies are in place to procure non-formulary alternatives when such circumstances arise that formulary options are unsuitable or have been exhausted [16, 17].

Such mechanisms that facilitate introducing non-formulary drugs (NFD) are not without challenges. Multiple studies have investigated the inappropriate prescribing of NFD with statistics indicating that up to 70% of NFD usage being inappropriate or not following the ascribed NFD policies [18–20]. The potential detrimental implications of prescribing NFD have been reported. These include: delays in initiating therapy due to lack of product availability; compromised medicine safety in prescribing, dispensing or administration errors due to clinician’s unfamiliarity; and undue financial-burden [18, 21–23].

However, research investigating the influencers of inappropriate prescribing of NFDs is lacking. The limited studies include a 2001 retrospective case–control study which was conducted in the Netherlands across three teaching hospitals which assessed patient, prescriber, drug, and drug formulary characteristics as potential indicators of non-adherence to drug formulary prescribing [24]. The study indicated that non-adherence was characterized by newly marketed drugs; drugs that were part of patients' pre-admission drug therapy; drugs where there are several other drugs within the same pharmacological class on the market; and drugs originating from a pharmacological class for which the formulary was highly restrictive. Furthermore, non-adherence was not correlated to medical specialty, therapeutic area, nor patient characteristics.

One further study conducted in the US in 2007 investigated pharmacists’ adherence to pharmacy and therapeutics (P&T) committee guidelines and policies. The study concluded ambiguous responsibility and low perceived importance as significant contributors to non-adherence [25].

The dearth of research to understand inappropriate prescribing of NFDs is in contrast to the abundant literature related to clinical practice guidelines (CPGs), which, like drug formularies, have been promoted as a means to enhance patient care, safety and mitigate against the increasing cost of health care through standardization of care [26]. Lack of knowledge, patient preference, and contraindications have been widely cited as reasons for their non-compliance [26–29]. Indeed, a recently published scoping review of CPG adherence studies that utilized behavioral theory to support their investigations, provides greater insights into the specific behavioral domains that have been most frequently associated with clinician’s CPG non-adherence [30].

The study reports that the behavioral theories adopted in many of the 12 studies included in the review are captured within the Theoretical Domains Framework (TDF), an integrative framework of 33 behavior change theories and 128 constructs, described in 14 domains [31]. The TDF domain of *environmental context and resources*, was reported to be most implicated in physician’s CPG non-adherence.

A similar scoping review investigating the implementation and use of guidelines among physicians, revealed that whilst a range of theories have been utilized, few studies had applied theory to evaluate interventions thereby limiting the interpretation and replication of interventions [32].

The authors of both studies note that the use of theory in such studies aids in the development and implementation of targeted interventions, which are more likely to yield sustained effectiveness rather than those developed more pragmatically. This is in accordance with recommendations of the United Kingdom (UK) Medical Research Council (MRC) guidance on ‘Developing and implementing complex interventions’ [33]. The UK MRC attributes the use of theory, specifically behavior change theories, as key to understanding barriers and exploring mediating pathways and moderators in designing interventions.

The TDF, being an overarching and comprehensive framework of behavioral theories, is thus being increasingly applied to investigations conducted in various settings and to study diverse clinical behaviors. The 14 TDF domains can act as behavioral change intervention targets, which may be mapped to specific evidence-based behavior change techniques. There is a growing body of evidence from the healthcare literature utilizing this approach to facilitate intervention development [34–39]. However, to our knowledge such studies have not been extended to investigate the behavioral influencers of prescribing non-formulary drugs.

Hamad Medical Corporation (HMC) is the main provider of secondary and tertiary healthcare in Qatar. Within HMC, the Pharmacy and Therapeutics Committee is mandated to develop, manage, monitor, update and administer the HMC Formulary. HMC policies and procedures provide transparent processes relating to: evaluating requests for new drugs in terms of clinical and cost effectiveness, patient safety, budget impact; removal of drugs from the formulary; and the follow-up and monitoring of new formulary drugs. The formulary is wide in scope, listing drugs within all therapeutic classes. There is a well-defined process for considering requests to for non-formulary drugs and to add drugs to the formulary.

The volume and cost of non-formulary drugs to HMC is increasing rapidly. While in 2013, the total cost of non-formulary drugs was 61,466,029 QAR, this had risen to 70,629,553 QAR for the period January to August 2016. This study, therefore, investigated physicians’ views and influences of prescribing NFDs within HMC.

### Aim

The study aimed to explore physicians’ views and influences on their prescribing of non-formulary drugs.

### Ethics approval

The study was granted ethics approval by HMC Medical Research Council (IRGC-06-JI-19-201 2 October 2020) and Qatar University (QU-IRB 1437-EA/20 6th December 2020). Prior to each interview, participants were reassured that their comments would remain confidential and reporting would be anonymised.

## Method

The study was designed, conducted and reported in accordance with the Consolidated Criteria for Reporting Qualitative Research (COREQ) [40].

### Study design

A mixed-methods exploratory sequential design was adopted in which data collection occurred in two phases. Phase one was a cross-sectional survey of prescribers, underpinned using the Theoretical Domains Framework (findings not reported in this manuscript) [41]. Survey respondents could opt-in to participate in this phase 2 qualitative study. The findings from phase one of this research informed the TDF domains where additional emphasis was place within the interview guide.

### Setting

The research was conducted within HMC, the main provider of secondary and tertiary healthcare in Qatar. HMC manages 12 hospitals, 9 specialist hospitals and 3 community hospitals, the national ambulance service and home and residential care services [42].

### Study participants, sampling and sample size

Physicians who submitted a non-formulary drug (NFD) request to the HMC Pharmacy and Therapeutics Committee via their hospital committee in the preceding 12 months were eligible to participate in the study. Participants were identified from the formulary management system records, with no exclusions. Purposive sampling was used, with physicians selected in strata of clinical specialty.

Sampling and data generation were continued to the point of data saturation, at which no new themes were generated from the data analysis. The approach to determining the point of saturation for qualitative interview studies recommended by Francis et al. was employed [43]. The initial analysis sample of 10 interviews was specified a priori, with a stopping criterion of 3.

### Interview schedule development and testing

A draft semi-structured interview schedule was developed from a related published review [30] and primary research study [41]. The interview schedule items were focused on prescribing NFDs and were derived from the 14 TDF overarching domains of behavioral determinants, i.e. influences (*knowledge, skills, social/professional role/identity, beliefs about capabilities, optimism, beliefs about consequences, reinforcement, intentions, goals, memory/attention/decision processes, environmental context/resources, social influences, emotions, behavioral regulation*) [44, 45]. Grounding the interview schedule in a theoretical framework added to the study credibility, ensuring that relevant domains and constructs were considered in data generation and analysis [33, 46]. A draft of the schedule was reviewed for credibility (coverage of the study aim) by six researchers from Qatar and Europe with expertise in qualitative research and use of TDF. Three pilot interviews were conducted by researchers (female graduate pharmacy students) trained in qualitative interviewing (TH, SA, NA) prior to finalizing the interview schedule. Senior researchers (DS, ZN) reviewed pilot interview recordings, providing feedback on interview approach, promoting credibility and dependability [47]. The pilot interviews were not included in the data analysis.

### Recruitment

Potential participants were identified by senior members of the Pharmacy and Therapeutics Committee and sent an e-mail on behalf of Executive Director of Pharmacy, with full study information. Those interestedwere requested to respond via email, following which they were contacted to organize the date and time of interview.

### Data generation

All participants provided signed, informed consent prior to the interview commencing. Interviews of around 45 min duration were conducted by the aforementioned researchers, in English via Microsoft Teams® between October and December 2021. The interviews were audio-recorded and transcribed in full, using a naturalistic approach in which every utterance was transcribed in as much detail as possible [48]. All interviewees were afforded the opportunity to review their transcripts prior to analysis. Furthermore, a very clear audit trail was maintained with documented details of data gathering, promoting dependability and reflexivity [47].

### Data analysis

Transcripts were analyzed using the framework approach of: data familiarization; identifying coding framework; indexing and charting; mapping; and interpreting [49].TDF was used as a coding framework for text relating to behavioral influences. Three researchers (NA, SA, TH) independently coded each interview, with consensus reached by discussion at a meeting of the research team. As per the Francis et al. approach, analysis was undertaken following completion of the tenth interview. A further 3 interviews were then conducted and analyzed. As no new themes were identified, saturation was deemed to have been achieved and no further interviews conducted [43]. Limited details are provided of each interviewee to protect anonymity.

## Results

Thirteen interviews of approximately 45 min duration were conducted with senior physicians (either consultants or senior specialists) in specialties of cardiology (n = 3), endocrinology (2), physical rehabilitation (2), anaesthesiology (1), neurology (1), psychiatry (1), pulmonology (1), rheumatology (1) and urology (1).

Three overarching themes were identified: providing evidence-based care for individual patients; influences of others; and formulary management issues. Subthemes are mapped to specific TDF domains. Table 1 presents the barriers and facilitators from the physicians’ interviews that were mapped to the TDF domains.

**Table 1:** The barriers and facilitators identified from the physicians’ interviews mapped to Theoretical Domains Framework 14 domains

Domains of the TDF	Barriers identified from the physicians’ interviews	Facilitators identified from the participants’ interviews
Beliefs about consequences		Physicians believed that prescribing NFDs can contribute towards improved patient outcomes
Professional role and identity		Physicians believed that as ‘experts’ they are justified to prescribe NFDs Physicians believed that their role requires them to deliver optimal patient care and this sometimes means prescribing NFDs Physicians believed that their role requires them to deliver the last evidence-based care and this sometime requires them to prescribed NFDs
Intentions		Physicians’ expressed their intention to prescribe drugs within the formulary whenever possible
Goals		Physicians described that in circumstances that they prescribe NFDs, it is to achieve better patient outcomes
Social influence	Physicians recognised that patients/patient’s relatives’ expectations influencing the decision to prescribe NFDs Physicians described that patients insisting to continue therapyinitiated at other institutions accounts for cases of NFD being prescribed	Physicians believed that their prescribing decisions are not influenced by pressure that may be exerted upon them from colleagues
Environmental context and resources	Physicians claimed that the formulary itself was not up-to-date in terms of evidence based practice and thus necessitating prescribing NFDs Physicians claimed that the formulary should be more flexible for specialists who manage complex patients and have expedited authority to prescribe NFDs	

*TDF* Theoretical Domains Framework, *NFDs* Non-Formulary Drugs

### Theme 1: providing evidence-based care for individual patients

#### Subtheme 1: intentions to prescribe within the formulary

All interviewees described their intent to prescribe from within the formulary, largely for reasons of availability and ease of patient access to a continuous supply of medicine,

*“My first option will be formulary medications, rather than non-formulary…availability is important. There should be no shortage in the treatment course... So, it will be better for the patient…”* [P5]

One interviewee commented on the obvious need for a non-formulary request process,

*“…I don’t think we will cover all the medicines available in the world in our formulary so the process will be needed as long as we use it in a rational and monitored way”* [P11]

#### Subtheme 2: goals of better patient outcomes

There were clear goals in prescribing NFDs, associated withmore effective and safer patient outcomes. These were often described in situations where patients had not responded to, or were experiencing debilitating adverse effects, fromtrials of multiple formulary drugs,

*“OK, so when I decide to prescribe non-formulary, I would have exhausted all the formulary medication that’s available…and because the patient is not responding to any of the formulary medication one after another or the patient is getting a side effect…”* [P7]

#### Subtheme 3: beliefs about consequences of better patient outcomes

Beliefs about consequences were strongly linked to goals of better outcomes. One physician described a patient with rheumatoid arthritis.

*“…very complicated rheumatoid arthritis patient that tried all the TNF blockers…she tried Il-6, she failed. She tried B cell depletion, she had side effects which she could not tolerate…and then we started her on JAK inhibitor…she lost the response after three months and then finally I asked for ubaticinib…which is non-formulary and she responded beautifully”* [P7]

#### Subtheme 4: professional role and identity as ‘specialist’

Interviewees often described themselves as ‘specialists’, being ‘up-to-date’, ‘aware of the latest evidence’ and ‘responsible’ for patient care which could necessitate the use of NFDs.

As one interviewee explained,

*“If the patient needs it* [non-formulary]*, why not?...we are there for the patient’s best...if that medicine* [non-formulary] *is not available, and they need it…When you are at the interface of the patient, you have to find a solution”* [P13]

All interviewees commented that only specialists should prescribe non-formulary drugs,

*“…so nobody else can do* [prescribe non-formulary]*, specialist should write the medication”* [P1].

One commented on being ‘excited’ on prescribing a new non-formulary drug,

*“There’s always new medications that sometimes if colleagues talk about it, you would be excited to prescribe it to the patient”* [P2]

### Theme 2: influences of others on non-formulary prescribing

#### Subtheme 1: Social influences—patients influencing non-formulary prescribing

All interviewees described at length the influence of the patient in decision-making. While the specialists made the final decision and signed the prescription, patients often had a major influence. Terms such as ‘insisting’, ‘definitely would influence’, ‘lot of pressure’ and ‘negative influences’ were used.

As one interviewee explained,

*“…there’s a lot of pressure from patients who read about it* [non-formulary] *or hear about the medicine, it’s a new medicine, exciting new medicine…and that puts us on the pressure…you might be you know pressured by patients to give them a non-formulary medicine. Ten percent of the time we are obliged to give a non-formulary medication*” [P2]

Many described patients insisting to continue non-formulary drugs initiated at other institutions, often overseas,

*“…because they think that non-formulary are more effective than formulary because they are bringing it from abroad from known centers and known doctors so they think they are more effective than formulary counterparts”* [P6]

Interviewees highlighted failed attempts to dissuade patients,

*“…when I talk to them and try to convince them to change, they refuse saying ‘I’ve tried all these and the one in the United States was better, you know’… you’re pushed into a corner”* [P4]

*“I tried to convince them of the alternatives that we have, explaining that it’s readily available, it is as good, but sometimes if they’re insisting or they’re taking it from outside, I have to give it to them”* [P2]

Less frequently, they described situations where they had convinced patients that the formulary options would be suitable,

*“…I had an open discussion with him. It took me 45 minutes and I gave him the pros and cons, why he's asking for this medication and I as a physician didn't think that it was yet an indication…maybe in the future he may need it, but not at this stage. And then he was convinced”* [P7]

On occasion, the influence was exerted by the patients’ relatives,

*“Patients would be passive, and they have a family member who is taking care of everything and they're taking the decision and sometimes they might affect our decision by insisting on something* [non-formulary] *in particular”* [P2]

#### Subtheme 2: social influences—other colleagues influencing non-formulary prescribing

The social influence on non-formulary prescribing from colleagues was also reported, albeit less frequently. While the patient was frequently involved in decision-making, other members of the healthcare team, including the clinical pharmacist, were sometimes consulted. On occasion, contact was made with international colleagues,

*“…we take the consequences as a team, the senior people in the team, and actually sometimes we email our international colleagues…For example, this patient, I asked a lot of international people…she failed everything…”* [P7]

### Theme 3: formulary management issues

#### Subtheme 1: environmental context and resource—formulary and formulary processes not sufficiently responsive

Many interviewees commented on the need to review the processes for adding new drugs to the formulary, particularly regarding drugs approved elsewhere,

*“There are hundreds of millions of people on these medications and yet they* [formulary management process] *request proof on efficacy. I am not the pharmaceutical company here, it’s approved everywhere in the world. I think the process is unnecessarily troublesome and difficult”* [P1]

It was noted by one participant that the formulary itself was not up-to-date in terms of evidence-based practice

*“I think the formulary itself should be up-to-date. We cannot ask people to provide the best possible care for their patients if your formulary is not up-to-date so you can feel guilty, compelled to say deprive the patient from one option that you think might be more suitable for them just because it was not introduced in the formulary”* [P3]

One interviewee noted that specialists manage complex patients hence should have the authority to prescribe non-formulary drugs not available to others,

*“We are writing a lot of non-formulary because we are seeing the responses in our patients if they failed other medications. We should have still room to have those medications available for the non-responders who are complicated…We are subspecialty, so we see everything complicated. We are not a general, general internal”* [P7]

## Discussion

### Statement of key findings

This qualitative study provided rich insights into the behavioral influencers associated with prescribing NFDs. The study findings identified three major themes implicated in prescribing NFDs namely: *Providing evidence-based care for individual patients*; *Influences of others on non-formulary prescribing*; and *Formulary management issues.* Furthermore, subthemes mapped to the TDF elucidated the association of the following domains of behavioral determinants: *environmental context and resources; social influences; professional role and identity; beliefs about consequences; goals; intentions.*

These findings may be used to identify key domains to target in interventions to improve prescribing behaviors.

This study adds to the limited literature reporting on the influencers of prescribing NFDs. These findings may be used to support the development of behavioral change intervention targets that aim to reduce the inappropriate prescribing of NFDs and thereby reduce potential risk of compromised patient safety and excess expenditures.

### Strengths and weaknesses

To our knowledge, this study is the first to adopt a qualitative approach to investigate physicians’ prescribing of NFDs. Moreover, the use of TDF to underpin this investigation is a key strength, allowing identification of key determinants of prescribing NFDs. There are, however, several limitations to the study, thus the findings should be interpreted with these limitations. Whilst exhaustive steps were taken in methodological rigor to advance the trustworthiness of the study findings, it should be noted that this study was conducted with physicians employed in secondary and tertiary care settings in Qatar, and the findings may lack transferability to other settings and countries. However, there are similarities in some of the findings with the limited published research and therefore it is likely that the findings will have significant resonance elsewhere. Furthermore, this study investigated the views of physicians who prescribe NFDs and was subject to non-participation bias from those physicians who opted not to participant. The views of other stakeholders [50], for example other members of the multidisciplinary team involved in prescribing decisions, were not sought; an extended participant sample may have provided further insights.

### Interpretation

Whilst drug formularies provide guidance on prescribing the most appropriate and evidence-based drugs, and direct use toward the most efficacious, safest, and cost-effective therapies, circumstances may arise in which prescribing NFDs may be necessary to deliver optimal evidence-based care to patients. This study reported such findings, mapped to the TDF domains of *goals, intentions* and *belief about consequences, where* study participants acknowledged the importance of adhering to formulary prescribing but are faced with the need to prioritize improving patient outcomes. Combined with participants’ belief that as experts in their field, their decisions to prescribe NFDs are justified.

This finding resonates with those of one previous study, which reported NFD use is characterized by: newly marketed drugs; patients' pre-admission drug therapy; drugs with many fellow drugs within the drug group; and drugs originating from a drug group for which the formulary was highly restrictive [24]. This raises important questions as to what level of coverage should drug formularies aim for, and how flexible should policies be in governing prescribing NFD.

The TDF domain *environmental context and resources*, which was linked to formulary and formulary processes being inadequately responsive has been discussed in the literature. Challenges associated with maintaining up-to-date formularies and their cost-effectiveness continues to be debated [16, 51–55]. What is clear, however, is the strong consensus that continuous monitoring of NFD prescribing and periodic review of trends are essential parts of formulary management [56]. Beyond this, recent studies have advocated for the transition to more relaxed formularies, with expansion of the number of formulary items within selected drug classes in order to avoid the increased workload burden associated with the process of prescribing NFDs [55, 57–59]. However, there is likely to be considerable resistance against this approach. An open formulary may lead to multiple therapeutic duplications in one drug class and may have training implications for inexperienced prescribers in safe and rational prescribing when starting clinical practice. Furthermore, an expanded formulary could lead to more medicine errors, and a larger inventory which will have cost implications [60, 61].

Also reported in the literature, and consistent with this study’s findings are clinicians’ reservations of drug formularies. Studies have recorded physicians’ views that drug formularies limit clinical autonomy, and have become overly focused on cost containment and creating needless hurdles for physicians and patients [8, 62].

Ethical dilemmas, such as the role of social influences in prescribers’ decision-making, that have been reported in similar studies relating to prescribing of antidepressants [63], opioids [64] and antimicrobials [50], also featured in the physicians’ responses. There are also numerous studies indicating patients’ role in clinicians’ non-adherence to clinical practice guidelines and its potential to result in suboptimal healthcare and delivery [28, 65–67]. However, this is the first study to shed light on the challenges physicians can face from patients and patients’ relatives to prescribe NFDs. Ethical dilemmas relating to the influence of limited resources, which have been reported elsewhere in the literature [50, 68], were not reported in this study.

By utilizing the TDF domains to identify the significant behavioral determinants, targets for interventions may be determined, as proposed by the UK Medical Research Council (MRC) [33]. The Behavior Change Wheel identifies which domains encourage optimal strategies and the development of interventions aligned with behavioral determinants [69]. For example, determinant of social influences of prescribing NFDs (patients influencing non-formulary prescribing) will require education and persuasion interventions.

### Further research

To effectively change prescribing behaviors regarding NFDs, the application of TDF will necessitate participation and empowerment from all levels of the organization. As a result, future studies should prioritize exploring these elements, utilizing rigorous mixed-method research approaches that encompass inclusion of all stakeholders involved including patients.

## Conclusion

This qualitative study has identified key behavioral determinants influencing prescribing non-formulary drugs, which were categorized within three main themes: providing evidence-based care for individual patients; influences of others on non-formulary prescribing; and formulary management issues.

The behavioral influencers can be mapped to behavior change strategies facilitating the development of an intervention to promote appropriate prescribing of NFDs with implications for medicine safety and healthcare efficiency.
